# Anti-Allergic and Anti-Inflammatory Effects of Neferine on RBL-2H3 Cells

**DOI:** 10.3390/ijms222010994

**Published:** 2021-10-12

**Authors:** Kuan-Ming Chiu, Yen-Ling Hung, Su-Jane Wang, Yi-Ju Tsai, Nan-Lin Wu, Cher-Wei Liang, Der-Chen Chang, Chi-Feng Hung

**Affiliations:** 1 Division of Cardiovascular Surgery, Cardiovascular Center, Far-Eastern Memorial Hospital, New Taipei City 22060, Taiwan; kmchius@gmail.com; 2 Department of Nursing, Oriental Institute of Technology, New Taipei City 22060, Taiwan; 3 Department of Photonics Engineering, Yuan Ze University, Taoyuan 32003, Taiwan; 4 Graduate Institute of Biomedical and Pharmaceutical Science, Fu Jen Catholic University, New Taipei City 24205, Taiwan; irishung0919@gmail.com (Y.-L.H.); med0003@mail.fju.edu.tw (S.-J.W.); 5 Graduate Institute, Department of Pharmacology, National Taiwan University College of Medicine, Taipei 10051, Taiwan; 6 School of Medicine, Fu Jen Catholic University, New Taipei City 24205, Taiwan; 065735@mail.fju.edu.tw (Y.-J.T.); 085027@mail.fju.edu.tw (C.-W.L.); 7 Department of Dermatology, MacKay Memorial Hospital, Taipei 104217, Taiwan; alvin.4200@mmh.org.tw; 8 Department of Medicine, Mackay Medical College, New Taipei City 25245, Taiwan; 9 Mackay Junior College of Medicine, Nursing, and Management, New Taipei City 25245, Taiwan; 10 Department of Mathematics and Statistics, Department of Computer Science, Georgetown University, Washington, DC 20057, USA; chang@georgetown.edu; 11 School of Pharmacy, Kaohsiung Medical University, Kaohsiung 80708, Taiwan

**Keywords:** neferine, natural product, mast cell, dermatitis, itching

## Abstract

Mast cells play a very important role in skin allergy and inflammation, including atopic dermatitis and psoriasis. In the past, it was found that neferine has anti-inflammatory and anti-aging effects on the skin, but its effect on mast cells has not yet been studied in detail. In this study, we used mast cells (RBL-2H3 cells) and mouse models to study the anti-allergic and inflammatory effects of neferine. First, we found that neferine inhibits the degranulation of mast cells and the expression of cytokines. In addition, we observed that when mast cells were stimulated by A23187/phorbol 12-myristate-13-acetate (PMA), the elevation of intracellular calcium was inhibited by neferine. The phosphorylation of the MAPK/NF-κB pathway is also reduced by pretreatment of neferine. The results of in vivo studies show that neferine can improve the appearance of dermatitis and mast cell infiltration caused by dinitrochlorobenzene (DNCB). Moreover, the expressions of barrier proteins in the skin are also restored. Finally, it was found that neferine can reduce the scratching behavior caused by compound 48/80. Taken together, our results indicate that neferine is a very good anti-allergic and anti-inflammatory natural product. Its effect on mast cells contributes to its pharmacological mechanism.

## 1. Introduction

Many studies have confirmed that allergic diseases are the result of not only innate immune activation, but also adaptive activation. Among them, mast cells play a very important role in a variety of allergic diseases such as allergic rhinitis, asthma, and allergic dermatitis. Mast cells express IgE receptors (FcεRI) on their cell membranes, and FcεRI receptors have high affinity for IgE antibodies [1,2]. When antigen-specific IgE binds to FcεRI, an interaction takes place and an allergic reaction occurs. The aggregation of FcεRI can activate receptor tyrosine kinases, initiate downstream reactions, and promote the phosphorylation of various key proteins (such as mitogen-activated protein kinase; MAPKs), ultimately leading to the influx of calcium ions (Ca^2+^), which is a key event in mast cell degranulation [3]. The activated mast cells degranulate and release chemical mediators. These chemical mediators used in allergic reactions can cause inflammation [4], so the treatment of allergic symptoms involves mast cell degranulation inhibitors. Therefore, the inhibition of IgE-mediated mast cell degranulation is often used to identify new compounds to prevent and treat allergic diseases. It has been established that anti-dinitrophenyl (DNP) IgE antibodies and antigens can induce passive skin hypersensitivity (PCA) reactions as a typical in vivo model of immediate hypersensitivity and as a research drug for the potential of anti-antigenic allergies [5,6]. In addition, mast cell degranulation can also be caused by non-immune stimulants, such as compound 48/80 and A23187. A23187, calcium ionophore, has long been known to stimulate mast cell secretion with the release of preformed mediators, such as histamine from their granules, serving as an example [4]. Compound 48/80 is a mixed polymer of p-methoxy-N-methylphenethylamine cross-linked by formaldehyde, which is widely used for IgE-independent stimulation of mast cells [7,8]. Therefore, appropriate amounts of compound 48/80 and A23187 have been commonly used as reagents to study the mechanism of allergic reactions. According to statistical evaluations, about 15–20% of the population suffers from atopic dermatitis. Whether it is children or adults who suffer from this disease, without proper treatment, atopic dermatitis can cause psychological distress to patients and burden their family and social health costs [9]. Unfortunately, there is no suitable treatment method to cure allergic dermatitis. Current treatment options still avoid disease-causing allergens, and continuous use of steroids are the gold standard of treatment. The inevitable allergens and the side effects of drugs have prompted the urgent development of new therapeutic drugs. Natural compounds isolated from medicinal herbs and plants are potential sources of therapeutic agents to prevent and treat inflammatory diseases and improve the quality of life for patients with allergic disorders.

Neferine comes from the lotus core (the embryo of the seed) of the plant *Nelumbo nucifera* and is a major bisbenzylisoquinoline alkaloid. There is evidence that neferine has a wide range of pharmacological properties, such as anti-cancer, anti-oxidant, anti-inflammatory, and neuroprotective effects, in many diseases [10,11,12,13,14,15]. Khan et al. found that neferine produces anti-photoaging effects by inhibiting the UV-mediated increase in ROS and malondialdehyde (MDA) levels in human keratinocytes and fibroblasts [16,17]. Recently, we found that neferine has potential as an alternative medicine for the treatment of atopic dermatitis or other skin-related inflammatory diseases [18]. However, whether neferine plays an anti-skin allergy effect in mast cells still remains uncertain. In this study, we aimed to investigate the effects of neferine on mast cell-mediated allergic diseases, using human mast cell lines (RBL-2H3 cells) and a compound 48/80- and DNCB-induced anaphylaxis mice model.

## 2. Results

### 2.1. Neferine Cytotoxicity Test on Rat Mast CelLs (RBL-2H3)

**Figure 1:**
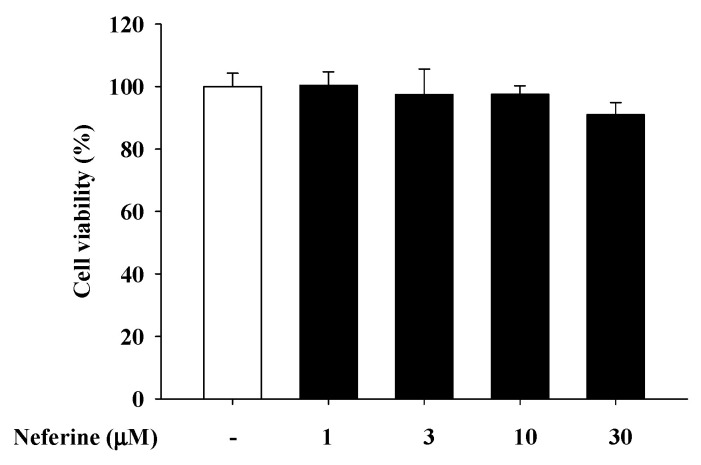
Cell viability in rat mast cells (RBL-2H3) under different concentrations of neferine treatment.

Since rat basophilic leukemia (RBL)-2H3 cells, a histamine-releasing mast cell analogue, are suitable cells to examine the effects of mast cell-mediated inflammation [19], we used these cells to investigate the anti-allergic effects of neferine. First, we tested whether neferine can produce cytotoxicity in mast cells. When the cells are pretreated with neferine from 1 to 30 μM for 24 h, the cell viability of the cells will not be affected by the treatment of neferine (Figure 1). 

### 2.2. Neferine Reduces the Degranulation Effect of RBL-2H3 Stimulated by Different Activators

**Figure 2:**
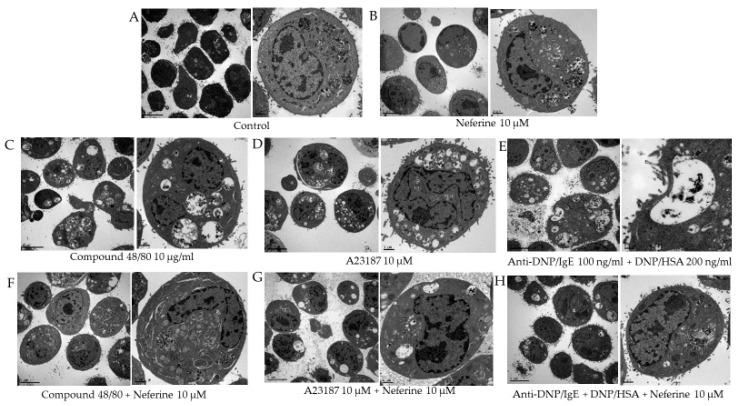
Degranulation of RBL-2H3 cells is induced by different activators and inhibited by neferine. (**A**) Control group. (**B**) RBL-2H3 cells are only treated with 10 μM neferine. (**C**–**E**) RBL-2H3 cells are only treated with compound 48/80 10 μg/mL for 30 min, A23187 10 μM for 10 min, and anti-DNP/IgE (100 ng/mL) for 24 h + DNP/HSA (200 ng/mL) for 1 h. (**F**–**H**) RBL-2H3 cells were pretreated with neferine 10 μM for 20 min and then various activators were added separately. The left panel is low magnification, and the right panel is high magnification.

RBL-2H3 cells are commonly utilized for in vitro studies of degranulation [20,21,22]. Stimulation of the RBL-2H3 cells with anti-dinitrophenol (DNP) IgE and DNP-conjugated human serum albumin (HSA) triggers degranulation. In addition, mast cells are known to degranulate in response to synthetic compounds, such as compound 48/80 and the calcium ionophore A23187, and these compounds have been used as convenient reagents for direct studies of the mechanisms of in vitro allergy responses [23,24]. The results showed that the cytoplasm of RBL-2H3 that was not activated by the activator contained limited secretory granules. Treatment of neferine (10 μM) alone did not affect the activity of RBL-2H3 (Figure 2A,B). However, the addition of different activators, including compound 48/80, A23187, and anti-DNP/IgE + DNP/HSA, clearly filled the cytoplasm with secretory granules. Under the electron microscope, it can be seen that there is a large number of secretory granules in the cytoplasm, and some secretory granules even fuse with the cell membrane, leading to degranulation (Figure 2C–E). After pretreatment with neferine 10 μM, the secretory granules in the cytoplasm were significantly reduced (Figure 2F–H). These results indicate that neferine can effectively inhibit the activation and degranulation of RBL-2H3 stimulated by the activator.

### 2.3. Neferine Inhibits PMA/A23187-Induced Intracellular Calcium Elevation in RBL-2H3

**Figure 3:**
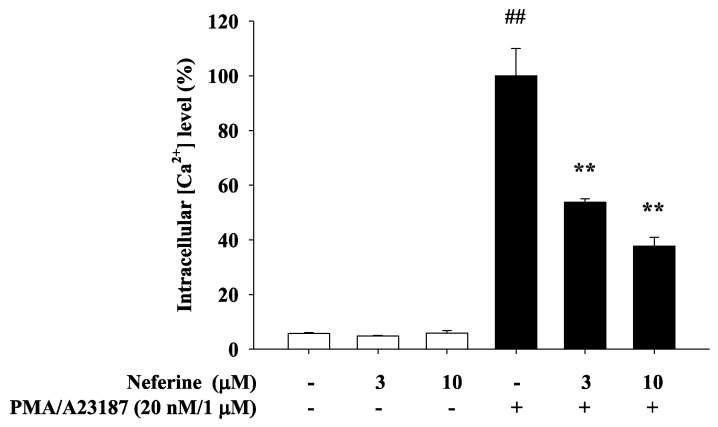
Neferine inhibits the effect of PMA/A23187 in rat mast cells (RBL-2H3) on the level of intracellular calcium. The [Ca^2+^]i elevation induced by PMA/A23187 in rat mast cells (RBL-2H3) was affected by neferine 3 μM and 10 μM for 20 min. Values represent the mean ± SEM from at least three experiments. ## *p* < 0.01 vs. control, ** *p* < 0.01 vs. negative control, PMA/A23187.

Calcium ions play the role of secondary messenger in the process of cell activation. A23187 is the most effective way to increase the penetration of calcium ions through cell membranes and stimulate the activation of mast cells. Phorbol myristate acetate (PMA) can activate protein kinase C and increase intracellular calcium ions to enhance the effect of A23187. The increase in calcium ions in mast cells has also been considered as an important factor in inducing mast cell activation, so that it affects the degranulation of mast cells and releases more inflammatory cytokines and chemokines. Therefore, this study sought to explore whether neferine can inhibit the calcium ion concentration in RBL-2H3 when it inhibits inflammation. The experimental group was divided into with or without neferine pretreatment for 20 min, and 1 mM CaCl_2_ and PMA/A23187 were added to stimulate and activate RBL-2H3. The experimental results showed that treatment of RBL-2H3 with different concentrations of neferine alone did not affect the increase in intracellular calcium ion concentration. In the PMA/A23187-induced Ca^2+^ influx group, the intracellular calcium ion concentration increased significantly. RBL-2H3 cells were pre-treated for 20 min with neferine (3 μM and 10 μM), and the calcium ion concentration was significantly reduced (Figure 3).

### 2.4. Neferine Reduced the mRNA Expression of Pro-Inflammatory Cytokines in PMA/A23187 Stimulated by RBL-2H3

**Figure 4:**
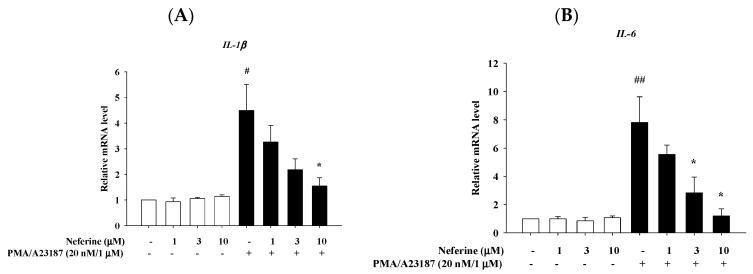

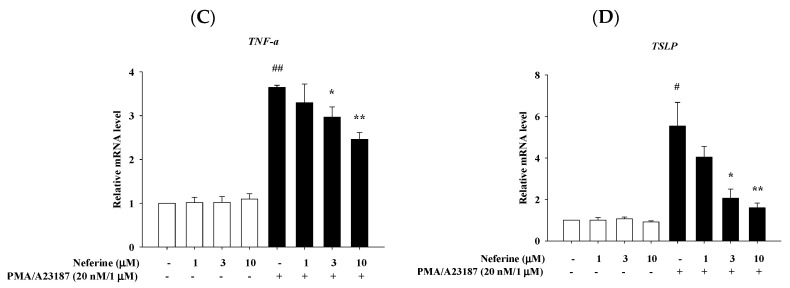
The effects of neferine on the mRNA expression levels of cytokines in PMA/A23187-stimulated RBL-2H3. RBL-2H3 cells were pretreated with different concentrations of neferine—1, 3, and 10 μM—for 20 min, and then the cells were treated with PMA/A23187 (20 nM/1 μM) for 6 h. Total RNA was isolated and mRNA expression level of (**A**) IL-1β, (**B**) IL-6, (**C**) TNF-α, and (**D**) TSLP were determined using qPCR. Values represent the mean ± SEM from the three independent experiments. # *p*< 0.05, ## *p* < 0.01 compared with the no-treatment condition, * *p* < 0.05, ** *p* < 0.01 compared with the only TNF-α/IFN-γ treatment condition.

The immune response of the skin will increase the number of mast cells in the body, and the mast cells will then produce pro-inflammatory cytokines and accelerate the progress of the disease. Pro-inflammatory cytokines, especially TNF-α, IL-6, IL-1β, and IL-8, not only induce inflammation but also cause leukocyte infiltration, granuloma formation, and tissue fibrosis. PMA/A23187 acts on mast cells and induces them to produce IL-1β, IL-6, TSLP, and TNF-α, causing allergy-related inflammation. Therefore, we pre-treated RBL-2H3 with neferine for 20 min and stimulated with PMA/A23187 for 6 h to detect whether neferine can down-regulate the mRNA expression of IL-1β, IL-6, TSLP, and TNF-α. The results showed that in the group treated with neferine alone, the expression of cytokine mRNA was not affected, and the expression of mRNA in the group that induced inflammation by PMA/A23187 increased significantly. In the group treated with neferine, it was observed that the mRNA expression levels of IL-1β, IL-6, TSLP, and TNF-α were significantly suppressed (Figure 4).

### 2.5. Neferine Inhibits PMA/A23187-Induced Phosphorylation of MAPK Pathway in RBL-2H3

**Figure 5:**
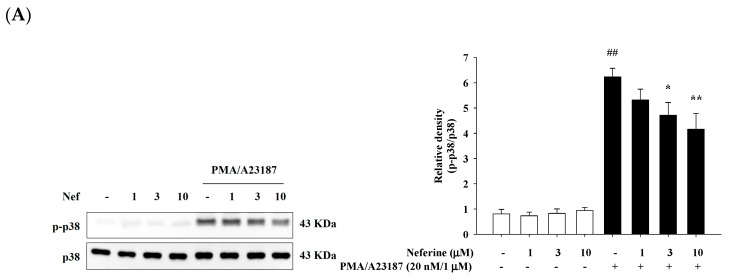

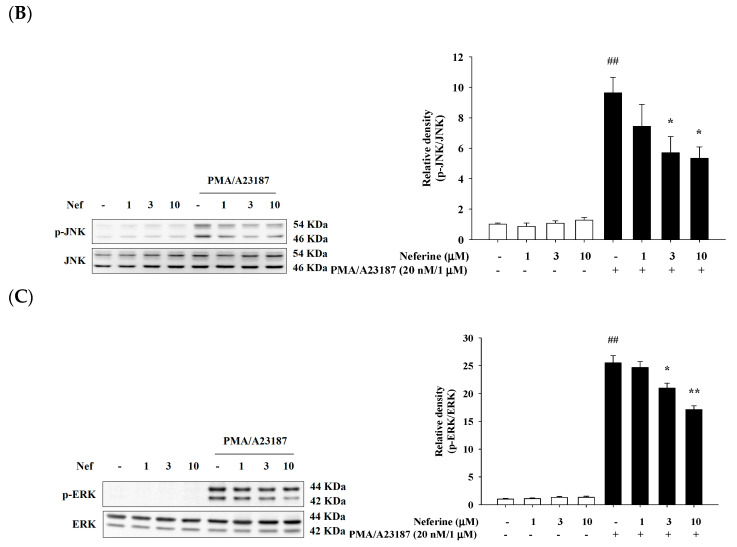
Neferine reduced PMA/A23187-induced JNK (**A**), p38 (**B**), and ERK (**C**) activation in RBL-2H3 cells. RBL-2H3 cells were pretreated with different concentrations of neferine—1, 3, and 10 μM—for 20 min, and then the cells were treated with PMA/A23187 (20 nM/1 μM) for 30 min. Western blots were analyzed quantitatively. Values represent the mean ± SEM from the three independent experiments. ## *p* < 0.01 compared with the no-treatment condition, * *p* < 0.05, ** *p* < 0.01 compared with the only PMA/A23187 treatment condition.

Previous studies have pointed out that mast cells need to release intracellular calcium ions to activate MAPK. In addition, the MAPK signaling pathway regulates the expression of monocytes, T lymphocytes, B lymphocytes, cytokines, and chemokines, and induces autoimmunity. This mechanism is similar to the activation pathway of PMA/A23187. In this study, we explored whether neferine can participate in the regulation of MAPK pathways. We treated different concentrations of neferine (1, 3, and 10 μM) to RBL-2H3 for 20 min and then stimulated with PMA/A23187 for 30 min to induce inflammation. The results showed that when RBL-2H3 was treated with different concentrations of neferine alone, the activation of p38, JNK, and ERK proteins was not affected. In the group treated with PMA/A23187 alone, we found that p38, JNK, and ERK proteins were activated and have significant phosphorylation. In the group pretreated with neferine, it was observed that for higher concentrations of neferine, the phosphorylation of p38, JNK, and ERK proteins was significantly reduced (Figure 5A–C).

### 2.6. Neferine Inhibits PMA/A23187-Induced Phosphorylation of NF-κB Pathway in RBL-2H3

**Figure 6:**
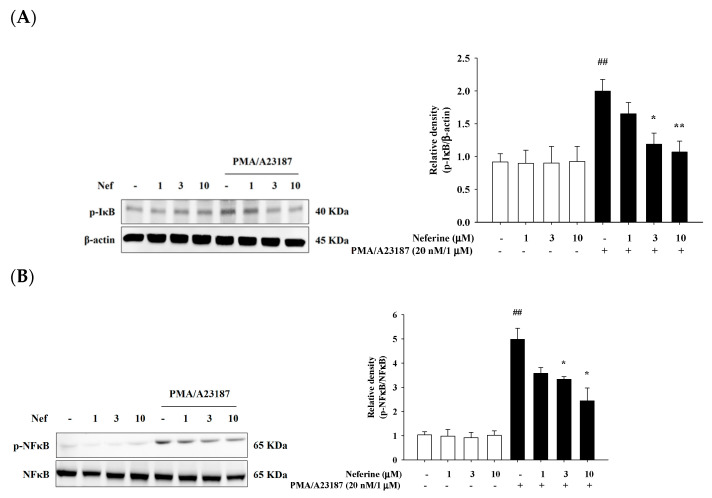
Neferine inhibited PMA/A23187-induced IκB (**A**) and NFκB (**B**) activation in HaCaT cells. Cells were pretreated with different concentrations of neferine—1, 3, and 10 μM—for 20 min and then treated with PMA/A23187 (20 nM/1 μM) for 30 min. *Western blots* were analyzed quantitatively. Values represent the mean ± SEM from the three independent experiments. ## *p* < 0.01 compared with the no-treatment condition, * *p* < 0.05, ** *p* < 0.01 compared with the only PMA/A23187 treatment condition.

The expression of pro-inflammatory cytokines depends on the activation of the transcription factor NF-κB in mast cells. When the IκB protein was phosphorylated and degraded, NF-κB was released and migrated into the nucleus, binding with its corresponding DNA responsive elements and promoting the transcription of proinflammatory mediators. Many cytokines and chemokines were produced, which regulate immune response, differentiation, and inflammation. Therefore, we explored whether pretreatment with neferine affects protein phosphorylation induced by PMA/A23187. We treated different concentrations of neferine (1, 3, and 10 μM) to RBL-2H3. After 20 min, PMA/A23187 stimulated RBL-2H3 for 1 h or 2 h to induce inflammation. The results showed that treatment of RBL-2H3 with different concentrations of neferine alone did not affect the phosphorylation of IκBα and NF-κB. When PMA/A23187 was used for stimulation, a significant increase in the phosphorylation of IκBα and NF-κB was observed. In the group pretreated with neferine, it was found that the phosphorylation of IκBα (Figure 6A) and NF-κB (Figure 6B) was significantly reduced.

### 2.7. Neferine’s Effect on 2,4-Dinitrochlorobenzene (DNCB) Induced Atopic Dermatitis

**Figure 7:**
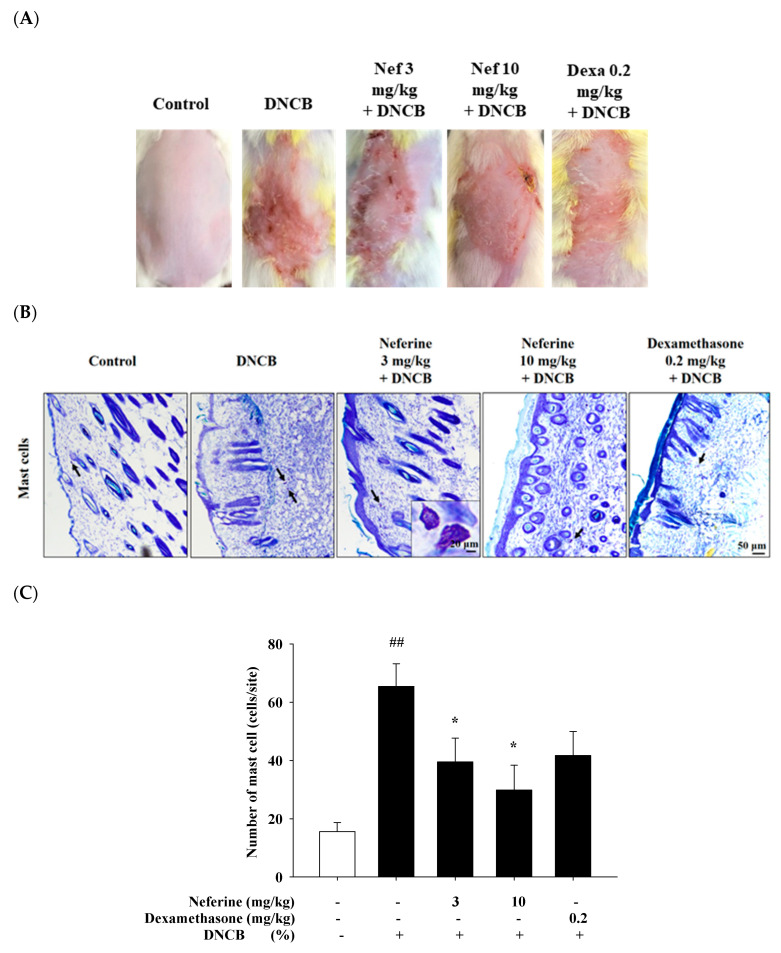
Histological analysis to assess the effect of neferine on atopic dermatitis (AD)-like skin lesions and mast cell infiltration after the 15th DNCB challenge. (**A**) Phenotypic presentation of mouse skin after 15 days treatment. (**B**) Toluidine blue staining; scale bar: 50 μm. (**C**) Number of mast cells. ## *p* < 0.01 compared with the no-treatment condition, * *p* < 0.05 compared with the only DNCB treatment condition.

An animal model for disease research, DNCB is a representative irritant that causes atopic dermatitis [25]. Our experiment uses 1% dinitrochlorobenzene (DNCB) dissolved in 75% alcohol to induce the first stage of sensitization response. Neferine (3 mg/kg and 10 mg/kg) and dexamethasone (Dexa, 0.2 mg/kg) dissolved in DMSO were administered intraperitoneally on the fifth day for ten days. On the 8th, 11th, and 14th days, 0.5% DNCB was applied as the second stage of inducing inflammation. Mice were divided into four groups—control group, DNCB group, neferine-treated group, and dexamethasone-treated group—and then, after comparison among the groups, the differences were noted. We observed that DNCB induced an atopic skin inflammation reaction on the skin of mice. After applying DNCB, it was found that the skin of the mice increased redness and scaling. These are all due to abnormal keratinocyte proliferation, infiltration, and aggregation of immune cells. The group treated with neferine 3 mg/kg and dexamethasone 0.2 mg/kg for four days had alleviated redness. We continued to observe the condition of the dorsal skin of the mice up to day 15. Compared with the control group, the DNCB group had severely red and swollen skin conditions, and there were dander and wounds that seemed to be caused by itching and pain. We can find a trend of decreasing degree after treatments of neferine and dexamethasone (Figure 7A). Toluidine blue stain was used to observe the number of mast cells aggregated. The mast cells appeared purple when observed under a microscope. It can be quantitatively shown that many mast cells infiltrated and accumulated in the dermis in the DNCB experimental group, significantly increased compared with the control group. In the group treated with neferine and dexamethasone, the infiltration, aggregation, and number of mast cells were decreased (Figure 7B,C).

### 2.8. Neferine Improves the mRNA Expression of Barrier-Related Molecules after Treatment of DNCB

**Figure 8:**
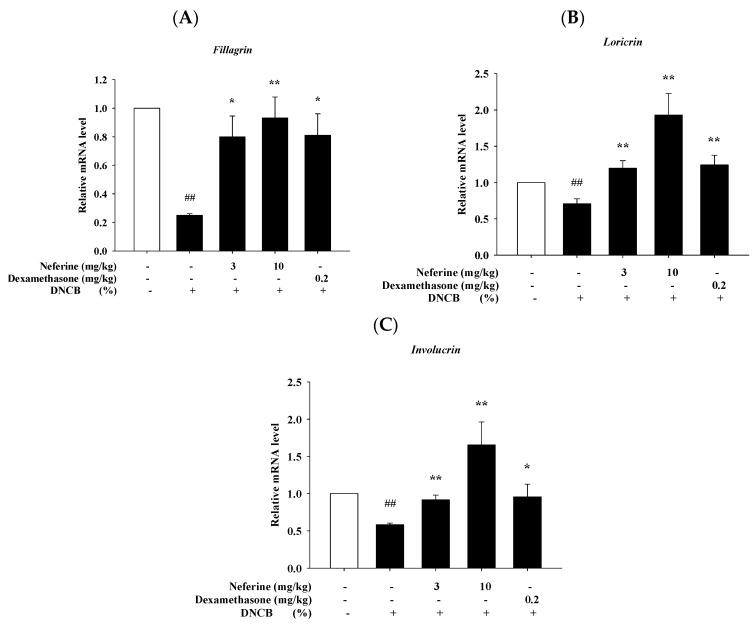
Effect of neferine on barrier-related molecules’ mRNA expression of atopic dermatitis-like phenotype in BALB/c mice. (**A**) Filaggrin, (**B**) loricrin, (**C**) involucrin. Values represent the mean ± SEM from the three independent experiments. ## *p* < 0.01 compared with the no-treatment condition, * *p* < 0.05, ** *p* < 0.01 compared with the only DNCB treatment condition.

The skin-related inflammation caused by AD can cause abnormal skin barrier function. If the skin barrier is defective, it is easier for antigens or irritants to penetrate the skin epidermis and enter the body to induce immune-related reactions. At the same time, the production of differentiation proteins, including filaggrin, loricrin, and involucrin, will be suppressed. Therefore, restoring the damaged skin barrier is very important for preventing and treating AD [26]. In our study, we used neferine to intervene in the animal model of DNCB-induced atopic dermatitis. After sacrificing mice, the dorsal skin tissue was grounded for RT-qPCR analysis to evaluate whether neferine can improve the mRNA expression reduction in filaggrin, loricrin, and involucrin after the treatment of DNCB. The results showed that the DNCB experimental group had significantly reduced mRNA expression of filaggrin, loricrin, and involucrin compared to the control group. The mRNA expression of filaggrin, loricrin, and involucrin was significantly increased in the group treated with neferine and dexamethasone compared to the DNCB group (Figure 8).

**Figure 9:**
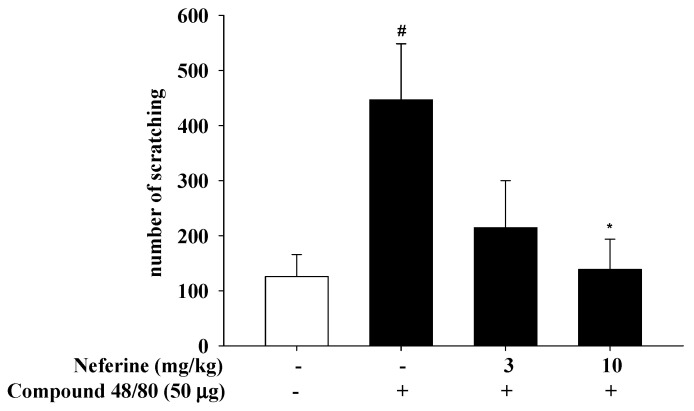
The effects of neferine for treatment of 5 days on compound 48/80-induced scratching behavior in BALB/c mice. Each column and vertical bar show the mean ± SEM from the three independent experiments. #, * *p* < 0.05 compared with the control group and compound 48/80 alone group (Student’s *t*-test).

We further proved that neferine has an anti-allergic scratching effect. We used a mast cell degranulation agent, compound 48/80, to induce scratching behavior in BALB/c mice. As shown in Figure 9, we found that the treatment of neferine inhibited the scratching behavior caused by compound 48/80 (50 μg/site). Repeated administration of lower and higher doses of neferine (3 and 10 mg/kg) for 5 consecutive days significantly reduced the number of scratches caused by compound 48/80.

## 3. Discussion

The pathogenesis of atopic dermatitis (AD) is not fully understood, but the disease is mediated by an abnormal immune globulin E (IgE) immune response in skin barrier dysfunction. Among them, mast cells (MC) can cause IgE-mediated allergic diseases, including AD. When mast cells are activated, they release their membrane-bound cytoplasmic particles, leading to the release of a variety of molecules. These molecules play an important role in the pathogenesis of AD and host defense [27]. Because inhibiting mast cell activation or degranulation helps to regulate various IgE-mediated hypersensitivity reactions, mast cells are one of the ideal targets for exploring anti-allergic drugs. In our study, mast cells are degranulated in response to compound 48/80 and the calcium ionophore A23187, and anti-DNP/IgE + DNP/HSA. We found that neferine effectively inhibited the degranulation effect of mast cells, showing its anti-allergic reaction. Moreover, the increase in intracellular calcium and activation of MAPK were inhibited by neferine. Furthermore, the skin mast cell infiltration caused by DNCB and the scratching behavior caused by compound 48/80 were also reduced. Therefore, the mechanism of neferine’s anti-atopic dermatitis has a multiple effect in keratinocytes and mast cells.

The cytoplasm of mast cells found in tissues is filled with large, dense particles of pre-formed inflammatory mediators [28]. The process of releasing these mediators from the secretory granules in mast cells is called degranulation. Since inhibiting mast cell degranulation can regulate various IgE-mediated hypersensitivity reactions, mast cells are one of the ideal targets for exploring anti-allergic drugs [29,30]. Our results show that neferine has a very good effect on the degranulation of mast cells regardless of the use of antigen, receptor stimulator, or ionophore. It shows that neferine is a potential anti-allergy agent (Figure 2).

All pre-stored granules, newly synthesized mediators, and even granules and their components can be used as MC activation indicators [31]. In addition, the standard method is the measurement of intracellular Ca^2+^ concentration. Most of the MC secretagogues (e.g., antigen, compound 48/80) that have been generally used in the laboratory caused an increase in the intracellular Ca^2+^ concentration. In fact, the increase in intracellular Ca^2+^ concentration can also trigger MC degranulation. Usually, the increase in intracellular Ca^2+^ is induced by activating MC with compounds, such as thapsigargin (specifically block sarco/endoplasmic Ca^2+^ ATPase, SERCA) pumps and ionophores (i.e., ionomycin and A23187) [32,33]. Therefore, in the Ca^2+^-dependent MC activation, the measurement of Ca^2+^ influx is also considered as a method to determine MC activation conditions. In MC research, Fura-2 are widely used for the determination of intracellular Ca^2+^ changes. In our experiments, we found that the fluorescence intensity of activated mast cells was significantly suppressed after being treated with neferine (Figure 3). Previous reports indicated that the increase in intracellular Ca^2+^ concentration was in accordance with the release of histamine [34]. Therefore, we infer that the release of histamine will also be inhibited by the treatment of neferine. Further experiments are warranted to verify this hypothesis.

Mast cells have significantly different functional outputs. These include degranulation, formation of arachidonic acid precursors, chemotaxis, and cytokine production, and are all dependent on the calcium signals to some extent, regardless of stimulants [32]. Activated MC releases cytokines, which are the main mediators of allergies and inflammatory diseases [35]. The production of cytokines by activated mast cells is the result of the induction of cytokine gene transcription in these cells. The stimulation of mast cells leads to the activation of NF-kB and AP-1 and the production of many cytokines [36]. MAPK cascade-signaling pathways play an essential role in the regulation of the expression of cytokines. The activation of these pathways through PKC’s activation by PI results in the activation of various genes, including inflammatory cytokine genes such as IL-1β, IL-6, and TNFα [37]. Our recent studies have shown that neferine can inhibit the cytokines released by the activation of keratinocytes. At the same time, we also found that the pathway activation of MAPK will also be inhibited by the treatment of neferine [18]. In our current study, we showed that neferine may also reduce the expression of cytokines through the inhibition of MAPK activation in mast cells (Figure 4 and Figure 5). The activation of NFκB is also reduced due to the inhibition of MAPK activation (Figure 6).

A number of studies have reported that a variety of cytokines in AD patients are elevated, leading to skin barrier dysfunction, including cytokines secreted by keratinocytes and Th2-derived cytokines [38,39]. In addition, it is reported that these secreted cytokines can also down-regulate the expressions of tight junction (TJ) proteins and terminal differentiation genes, including filaggrin (*FLG*), loricrin (*LOR*), and involucrin (*IVL*) [39,40]. (Pro)filaggrin expression is decreased in AD and is reversely associated with MC tryptase and IL-6 [41]. IL-1β mediates chronic inflammation in mice with an impaired skin barrier [35]. In previous study, it was reported that the expressions of *FLG*, *IVL*, and *LOR* were downregulated after application of DNCB on the dorsal skin of NC/Nga mice [42,43]. In the present study, the expressions of *FLG*, *IVL*, and *LOR* were considerably reduced in the dorsal skin of the control group, which indicated that the epidermal barrier was impaired. The administration of neferine significantly recovered decreased expressions of *FLG*, *IVL*, and *LOR* after treatment of DNCB, while neferine administration slightly increased the expressions of *IVL* and *LOR* compared with the control group (Figure 8).

It is known that compound 48/80 is an effective activator of skin mast cells. The skin response stimulated by compound 48/80 may induce scratching behavior by releasing histamine from mast cells [44]. Histamine released from activated mast cells plays an important role in the increase in vascular permeability caused by compound 48/80. During human pruritus, histamine released from mast cells through various stimuli is also considered to be an important mediator. Therefore, inhibiting degranulation of mast cells is an important step in regulating histamine release during scratching behavior. In this study, compound 48/80 caused effective activation of skin mast cells. However, pretreatment with neferine significantly reduced the degranulation level of mast cells (Figure 2). These results indicate that the anti-scratch behavioral effect of neferine may be due to the reduction in vascular permeability by regulating mast cell degranulation (Figure 9).

It is generally established that AD patients suffer from skin barrier dysfunction, skin inflammation, or both, so it is difficult to find an appropriate treatment method. Therefore, combination therapy is usually recommended. Our recent research has proved that neferine has immunomodulatory function and barrier repair ability. Therefore, an important feature of neferine in AD treatment in the future is the maintenance of skin function and improvement of skin hydration and barrier repair. Neferine can also reduce scratching behavior induced by allergens and irritants. Moreover, atopic march is a phenomenon-related disease that occurs and progresses step by step. Atopic dermatitis is often seen in infancy. Food allergy often occurs after 1–2 years of age. Allergic rhinitis can often start before and after school. Asthma symptoms appear at different times, but most of them are after allergic rhinitis. After childhood, atopic dermatitis and certain food allergies may improve or disappear. Allergic rhinitis and asthma are not easy to cure [45,46]. Therefore, they are comorbid phenomena in diseases. Natural products including neferine have the therapeutic potential of comorbidity, and their effectiveness in related diseases is worth studying in the future.

## 4. Materials and Methods

### 4.1. Rat Basophilic Leukemia Cells

Rat basophilic leukemia cells (RBL-2H3) were a gift from T.L. Hwang, Chang Gung University, Taoyuan, Taiwan. RBL-2H3 cells are mucosal mast cells that are used to study antigen or calcium ionophore-induced degranulation, intracellular Ca^2+^, and signal transduction [29]. The cells were cultured in EMEM containing 10% FBS, with penicillin and streptomycin at 37 °C, 5% CO_2_. Iscove’s Modified Dulbecco’s Medium (IMDM) and fetal bovine serum (FBS) were purchased from Thermo Fisher Scientific (GIBCO^TM^, New York, NY, USA).

### 4.2. MTT Assay

3-(4,5-dimethylthiazol-2-yl)-2,5-diphenyl-2H-tetrazolium bromide (MTT) assay is used to measure the metabolic activity of cells. RBL-2H3 cells were seeded in a 24-well culture plate at 5 × 10^4^/well. After 24 h of drug treatment, 300 μL MTT per well was added and placed at 37 °C cell incubator for 2–4 h, then purple formazan crystals were dissolved with DMSO. An ELISA reader was used to measure the absorbance intensity at a wavelength of 550 nm as a cell viability test.

### 4.3. Measurement of Intracellular Ca^2+^ Level

[Ca^2+^]i was measured with fura-2 as described previously [47]. RBL-2H3 cells were resuspended in IMDM containing 5 μM Fura 2-AM and 1.2 mM CaCl_2_ at 37 °C for 30 min. After Fura-2 loading, keratinocytes were pelleted and resuspended in fresh DMEM. An aliquot (2 mL) was transferred to a stirred cuvette containing 1.2 mM CaCl_2_. Fura-2-Ca fluorescence was assayed at the excitation wavelengths of 340 and 380 nm (emission wavelength, 505 nm) in a PerkinElmer LS-55 spectrofluorometer (PerkinElmer Life and Analytical Sciences). Data were recorded at 5 s intervals [47].

### 4.4. Quantitative Polymer Chain Reaction (qPCR)

RBL-2H3 cells were planted in a 3.5 cm culture dish. After the cells grew to 90% full, the cells were grown in a static state for 24 h. After the cells were pretreated with neferine for 20 min, they were stimulated with TNF-α/IFN-γ for 1 or 24 h, respectively. The cells were scraped off, centrifuged (16,000× *g*, 10 min, 4 °C), and the supernatant was extracted. RNA was purified using total RNA isolation kit (GeneDireX^®^, Vegas, NV, USA). According to the operating procedure of iScript™ cDNA Synthesis Kit (BIO-RAD), reagents were added in order and operated according to the indicated conditions to convert RNA into cDNA. Furthermore, PowerUp™ SYBR™ Green Master Mix (Applied Biosystems™) was used. A total of 7.5 μL of ddH2O, 2 μL of cDNA, 0.25 μL of forward and reverse primers, and 10 μL of SYBR GREEN were added and mixed uniformly. The primer sequences are shown in Table 1 and Table 2. Then, RNA was quantified using ABI StepOnePlus™ Real-time PCR System.

### 4.5. Western Blot Assay

Western blotting is used to analyze the changes of various proteins in cells. RBL-2H3 cells were seeded in a 3.5 cm culture dish. After the cells grew to 90% full and were starved for 24 h, they were pretreated with neferine for 20 min, and then stimulated with TNF-α or IFN-γ for 30 min or 1 h, respectively. After scraping, they were pulverized by ultrasound and centrifuged (13,200 rpm, 10 min, 4 °C). After centrifugation, the supernatant was taken and the protein was quantified with a Pierce protein assay kit (Pierce, Rockford, IL, USA). It was electrophoresed on 10% SDS-polyacrylamide gel and then electroporated with PVDF membrane. After the transfer was completed, the PVDF membrane was put into TBS-T (Tris-buffered saline/0.05% tween 20) solution containing 5% skimmed milk powder and shaken for 1 h to avoid non-specific binding. Then, TBS-T was used to wash 3 times, 10 min each time. Then, primary antibodies (1:1000 dilution) were added and placed at 4 °C overnight and were then washed 3 times with TBS-T every 10 min. After adding secondary antibodies (diluted 1:1000) for 1 h, the PVDF membrane was washed 3 times with TBS-T for 10 min each time. Finally, the developer was added, and the membrane was placed in a chemical luminescence extraction system (BIOSTEP Celvin^®^) for shooting.

### 4.6. Dinitrochlorobenzene (2, 4-Dinitrochlorobenzene, DNCB) Induced Atopic Dermatitis-like Skin Inflammation in Mice

First, the mice were divided into four groups: control group, DNCB group (negative group), neferine (3 mg/kg and 10 mg/kg) with DNCB group, and dexamethasone (0.2 mg/kg) with DNCB group (positive group). Dexamethasone is a corticosteroid hormone that can reduce swelling and allergic reactions. In this in vivo experiment, both neferine and dexamethasone were dissolved in DMSO, while DNCB was dissolved in 75% ethanol by ultrasonic shock. The former were given by intraperitoneal injection, and for the latter, 100 µL was applied to the back skin and 20 µL was applied to the right ear. Three days before the experiment, the mice were anesthetized and the back hair was removed, and a small measuring magnet (SCT-MAG-TF) was embedded on the back of the mouse’s back feet. After resting for three days, it was confirmed that the mice were in good physical condition and the skin on the dorsal depilation area was normal, and the experiment was started. During the experiment, photographs were taken to record the appearance changes of the skin. The first stage (1–4 days) was the period of allergic atopic dermatitis. After measuring the basic values of mice in the DNCB group, the neferine (3 and 10 mg/kg) and DNCB experimental group, and the dexamethasone 0.2 mg/kg and DNCB experimental group, 1% DNCB was evenly applied to the back skin and right ear. On the fifth day, the drugs neferine and dexamethasone were injected intraperitoneally. The second stage (day 5 to day 14) was the re-induction of atopic dermatitis. In the 3 experimental groups, 0.5% DNCB was evenly smeared on the back skin and right ear of the mice. The next day, skin’s physiological values and pictures were taken and recorded. After the mice were euthanized with excessive carbon dioxide (CO_2_) on the 15th day, the dorsal skin tissue was removed for subsequent experimental analysis.

### 4.7. Compound 48/80-Induced Scratching Behavior in BALB/c Mice

The hair of the skin on the back of the mouse was cut, and 20 μL of compound 48/80 solution intracutaneously was injected. The control mice received saline injections instead. Immediately after the injection, the animal was placed in an observation cage (diameter 11 cm, MicroAct, Neuroscience, Tokyo, Japan), and the mouse’s scratching behavior was automatically and objectively detected and evaluated. The scratch behavior was measured for 60 min. MicroAct uses the following analysis parameters to detect waves corresponding to the continuous scratching behavior of mice: threshold, 0.05 V; event gap, 0.05 s; minimum duration, 0.25 s; maximum frequency, 30 Hz; minimum frequency, 5 Hz.

### 4.8. Statistical Analysis

Sigma-Plot software (Version 10.0) was used for the statistical analysis. Data are expressed as mean ± SEM, with (*) and (#) as notes. The statistical significance of the data was analyzed by unpaired, two-tailed Student *t*-tests. *p* values less than 0.05 and 0.01 were considered significant.

## 5. Conclusions

In this study, we determined that neferine effectively inhibits the degranulation of mast cells and reduces the expression of pro-inflammatory cytokines, which can reduce atopic dermatitis-like symptoms, restore the skin barrier, and reduce skin scratching. We have further proved that neferine can not only act on the keratinocytes of the skin, but also affect the activation of mast cells. It has more potential for application in allergic and inflammatory skin diseases.

## Figures and Tables

**Table 1:** Rat primer sequences for RT-qPCR.

Genes	Primers	Sequence (5′-3′)
IL-1β	Forward	CAG CTT TCG ACA GTG AGG AGA
	Reverse	TTG TCG AGA TGC TGC TGT GA
IL-6	Forward	ACA AGT CCG GAG AGG AGA CT
	Reverse	TTG CCA TTG CAC AAC TCT TTT C
TNF-α	Forward	ATGGGCTCCCTCTCATCAGT
	Reverse	GAAATGGCAAATCGGCTGAC
TSLP	Forward	TCA GGC AAC AGC ATG GTT CT
	Reverse	AAG TTA GTG CCA GCC GTA CC
GAPDH	Forward	TTC ACC ACC ATG GAG AAG GC
	Reverse	GGC ATG GAC TGT GGT CAT GA

**Table 2:** Mouse primer sequences for RT-qPCR.

Genes	Primers	Sequence (5′-3′)
IL-1β	Forward	TGG ACC TTC CAG GAT GAG GAC A
	Reverse	GTT CAT CTC GGA GCC TGT AGT G
IL-6	Forward	AGT TGC CTT CTT GGG ACT GA
	Reverse	TCC ACG ATT TCC CAG AGA AC
TNF-α	Forward	GGT GCC TAT GTC TCA GCC TCT TTT
	Reverse	GCC ATA GAA CTG ATG AGA GGG AG
TSLP	Forward	AGC TTG TCT CCT GAA AAT CGA G
	Reverse	AGG TTT GAT TCA GGC AGA TGT T
Fillagrin	Forward	ATG TCC GCT CTC CTG GAA AG
	Reverse	TGG ATT CTT CAA GAC TGC CTG TA
Involucrin	Forward	ATG TCC CAT CAA CAC ACA CTG
	Reverse	TGG AGT TGG TTG CTT TGC TTG
Loricrin	Forward	CTC CTG TGG GTT GTG GAA AGA
	Reverse	TGG AAC CAC CTC CAT AGG AAC
GAPDH	Forward	ACC CAG AAG ACT GTG GAT GG
	Reverse	CAC ATT GGG GGT AGG AAC AC

## References

[1] Gilfillan A.M., Beaven M.A. (2011). Regulation of Mast Cell Responses in Health and Disease. Crit. Rev. Immunol..

[2] Stone K.D., Prussin C., Metcalfe D.D. (2010). IgE, mast cells, basophils, and eosinophils. J. Allergy Clin. Immunol..

[3] Beaven M.A., Rogers J., Moore J.P., Hesketh T.R., Smith G.A., Metcalfe J.C. (1984). The mechanism of the calcium signal and correlation with histamine release in 2H3 cells. J. Biol. Chem..

[4] Moon T.C., Befus A.D., Kulka M. (2014). Mast cell mediators: Their differential release and the secretory pathways involved. Front. Immunol..

[5] Yoo J.M., Sok D.E., Kim M.R. (2014). Anti-allergic action of aged black garlic extract in RBL-2H3 cells and passive cutaneous anaphylaxis reaction in mice. J. Med. Food.

[6] Kono R., Nomura S., Okuno Y., Kagiya T., Nakamura M., Utsunomiya H., Ueno M. (2020). Two Japanese pepper (Zanthoxylum piperitum) fruit-derived compounds attenuate IgE-mediated allergic response in vitro and in vivo via inhibition of mast cell degranulation. Eur. J. Pharm..

[7] McNeil B.D., Pundir P., Meeker S., Han L., Undem B.J., Kulka M., Dong X. (2015). Identification of a mast-cell-specific receptor crucial for pseudo-allergic drug reactions. Nature.

[8] Ogasawara T., Murakami M., Suzuki-Nishimura T., Uchida M.K., Kudo I. (1997). Mouse bone marrow-derived mast cells undergo exocytosis, prostanoid generation, and cytokine expression in response to G protein-activating polybasic compounds after coculture with fibroblasts in the presence of c-kit ligand. J. Immunol..

[9] Avena-Woods C. (2017). Overview of atopic dermatitis. Am. J. Manag. Care.

[10] Xie J., Chen M.H., Ying C.P., Chen M.Y. (2020). Neferine induces p38 MAPK/JNK1/2 activation to modulate melanoma proliferation, apoptosis, and oxidative stress. Ann. Transl. Med..

[11] Zhong Y., He S., Huang K., Liang M. (2020). Neferine suppresses vascular endothelial inflammation by inhibiting the NF-κB signaling pathway. Arch. Biochem. Biophys..

[12] Tang Y.-S., Zhao Y.-H., Zhong Y., Li X.-Z., Pu J.-X., Luo Y.-C., Zhou Q.-L. (2019). Neferine inhibits LPS-ATP-induced endothelial cell pyroptosis via regulation of ROS/NLRP3/Caspase-1 signaling pathway. Inflamm. Res..

[13] Zhao L., Wang X., Chang Q., Xu J., Huang Y., Guo Q., Zhang S., Wang W., Chen X., Wang J. (2010). Neferine, a bisbenzylisoquinline alkaloid attenuates bleomycin-induced pulmonary fibrosis. Eur. J. Pharmacol..

[14] Marthandam Asokan S., Mariappan R., Muthusamy S., Velmurugan B.K. (2018). Pharmacological benefits of neferine - A comprehensive review. Life Sci..

[15] Yeh K.C., Hung C.F., Lin Y.F., Chang D.C., Pai M.S., Wang S.J. (2020). Neferine, a bisbenzylisoquinoline alkaloid of Nelumbo nucifera, inhibits glutamate release in rat cerebrocortical nerve terminals through 5-HT1A receptors. Eur. J. Pharmacol..

[16] Khan A., Bai H., Shu M., Chen M., Khan A., Bai Z. (2018). Antioxidative and antiphotoaging activities of neferine upon UV-A irradiation in human dermal fibroblasts. Biosci. Rep..

[17] Khan A., Bai H., Liu E., Chen M., Yu C., Wang R., Khan A., Bai Z. (2018). Protective effect of neferine against UV-B-mediated oxidative damage in human epidermal keratinocytes. J. Dermatol. Treat..

[18] Yang C.-C., Hung Y.-L., Ko W.-C., Tsai Y.-J., Chang J.-F., Liang C.-W., Chang D.-C., Hung C.-F. (2021). Effect of Neferine on DNCB-Induced Atopic Dermatitis in HaCaT Cells and BALB/c Mice. Int. J. Mol. Sci..

[19] Bae Y., Lee S., Kim S.H. (2011). Chrysin suppresses mast cell-mediated allergic inflammation: Involvement of calcium, caspase-1 and nuclear factor-κB. Toxicol. Appl. Pharm..

[20] Passante E., Frankish N. (2009). The RBL-2H3 cell line: Its provenance and suitability as a model for the mast cell. Inflamm. Res..

[21] Falcone F.H., Wan D., Barwary N., Sagi-Eisenberg R. (2018). RBL cells as models for in vitro studies of mast cells and basophils. Immunol. Rev..

[22] Weatherly L.M., Nelson A.J., Shim J., Riitano A.M., Gerson E.D., Hart A.J., De Juan-Sanz J., Ryan T.A., Sher R., Hess S.T. (2018). Antimicrobial agent triclosan disrupts mitochondrial structure, revealed by super-resolution microscopy, and inhibits mast cell signaling via calcium modulation. Toxicol. Appl. Pharmacol..

[23] Verbsky J.W., McAllister P.K., Malone D.G. (1996). Mast cell activation in human synovium explants by calcium ionophore A23187, compound 48/80, and rabbit IgG anti-human IgE, but not morphine sulfate. Inflamm. Res..

[24] Koch G., Habermann B., Mohr C., Just I., Aktories K. (1992). ADP-ribosylation of rho proteins is inhibited by melittin, mast cell degranulating peptide and compound 48/80. Eur. J. Pharm..

[25] Kim D., Kobayashi T., Nagao K. (2019). Research Techniques Made Simple: Mouse Models of Atopic Dermatitis. J. Investig. Dermatol..

[26] Kim H., Shin J.U., Lee K.H. (2013). Atopic dermatitis and skin barrier dysfunction. Allergy Asthma Respir. Dis..

[27] Nakamura Y., Oscherwitz J., Cease K.B., Chan S.M., Muñoz-Planillo R., Hasegawa M., Villaruz A.E., Cheung G.Y.C., McGavin M.J., Travers J.B. (2013). Staphylococcus δ-toxin induces allergic skin disease by activating mast cells. Nature.

[28] Gilfillan A.M., Tkaczyk C. (2006). Integrated signalling pathways for mast-cell activation. Nat. Rev. Immunol..

[29] Barsumian E.L., Isersky C., Petrino M.G., Siraganian R.P. (1981). IgE-induced histamine release from rat basophilic leukemia cell lines: Isolation of releasing and nonreleasing clones. Eur. J. Immunol.

[30] Naal R.M.Z.G., Tabb J., Holowka D., Baird B. (2004). In situ measurement of degranulation as a biosensor based on RBL-2H3 mast cells. Biosens. Bioelectron..

[31] Sahid M.N.A., Kiyoi T. (2020). Mast cell activation markers for in vitro study. J. Immunoass. Immunochem..

[32] Ma H.T., Beaven M.A. (2009). Regulation of Ca2+ signaling with particular focus on mast cells. Crit. Rev. Immunol..

[33] Ali H., Maeyama K., Sagi-Eisenberg R., Beaven M.A. (1994). Antigen and thapsigargin promote influx of Ca2+ in rat basophilic RBL-2H3 cells by ostensibly similar mechanisms that allow filling of inositol 1,4,5-trisphosphate-sensitive and mitochondrial Ca2+ stores. Biochem. J..

[34] Maeyama K., Sasaki M., Watanabe T. (1991). Simultaneous determination of intracellular calcium concentration and histamine secretion in rat basophilic leukemia cells (RBL-2H3). Anal. Biochem..

[35] Schwartz C., Moran T., Saunders S.P., Kaszlikowska A., Floudas A., Bom J., Nunez G., Iwakura Y., O’Neill L., Irvine A.D. (2019). Spontaneous atopic dermatitis in mice with a defective skin barrier is independent of ILC2 and mediated by IL-1β. Allergy.

[36] Kritas S.K., Saggini A., Varvara G., Murmura G., Caraffa A., Antinolfi P., Toniato E., Pantalone A., Neri G., Frydas S. (2013). Impact of Mast Cells on the Skin. Int. J. Immunopathol. Pharmacol..

[37] Zarubin T., Han J. (2005). Activation and signaling of the p38 MAP kinase pathway. Cell Res..

[38] Czarnowicki T., Krueger J.G., Guttman-Yassky E. (2014). Skin barrier and immune dysregulation in atopic dermatitis: An evolving story with important clinical implications. J. Allergy Clin. Immunol. Pract..

[39] Furue M. (2018). T helper type 2 signatures in atopic dermatitis. J. Cutan. Immunol. Allergy.

[40] Brunner P.M., Guttman-Yassky E., Leung D.Y.M. (2017). The immunology of atopic dermatitis and its reversibility with broad-spectrum and targeted therapies. J. Allergy Clin. Immunol..

[41] Ilves T., Tiitu V., Suttle M.M., Saarinen J.V., Harvima I.T. (2015). Epidermal Expression of Filaggrin/Profilaggrin Is Decreased in Atopic Dermatitis: Reverse Association With Mast Cell Tryptase and IL-6 but Not With Clinical Severity. Dermatitis.

[42] Lee J.W., Wu Q., Jang Y.P., Choung S.Y. (2018). Pinus densiflora bark extract ameliorates 2,4-dinitrochlorobenzene-induced atopic dermatitis in NC/Nga mice by regulating Th1/Th2 balance and skin barrier function. Phytother. Res..

[43] Kim, Seong, Choung (2020). Fermented Morinda citrifolia (Noni) Alleviates DNCB-Induced Atopic Dermatitis in NC/Nga Mice through Modulating Immune Balance and Skin Barrier Function. Nutrients.

[44] Inagaki N., Igeta K., Kim J.F., Nagao M., Shiraishi N., Nakamura N., Nagai H. (2002). Involvement of unique mechanisms in the induction of scratching behavior in BALB/c mice by compound 48/80. Eur. J. Pharm..

[45] Čelakovská J. (2015). Atopic march, food allergy and food hypersensitivity in children and adolescents suffering from atopic dermatitis. Food Agric. Immunol..

[46] Čelakovská J., Bukač J., Cermákova E., Vaňková R., Skalská H., Krejsek J., Andrýs C. (2021). Analysis of Results of Specific IgE in 100 Atopic Dermatitis Patients with the Use of Multiplex Examination ALEX2—Allergy Explorer. Int. J. Mol. Sci..

[47] Su I.C., Hung C.F., Lin C.N., Huang S.K., Wang S.J. (2019). Cycloheterophyllin Inhibits the Release of Glutamate from Nerve Terminals of the Rat Hippocampus. Chem. Res. Toxicol..

